# Non hepatitis jaundice - adverse reaction to rifampicin

**DOI:** 10.1186/1471-2334-14-S7-P48

**Published:** 2014-10-15

**Authors:** Ruxandra Laza, Rodica Potre Oncu, Alexandru Crişan, Ruxandra Jurac

**Affiliations:** 1 Dr. Victor Babeş University of Medicine and Pharmacy, Timişoara, Romania; 2 Dr. Victor Babeş Clinical Hospital of Infectious Diseases and Pneumology, Timişoara, Romania; 3 Clinical Emergency Children’s Hospital “Dr. L. Turcanu”, Timişoara, Romania

## Background

The hopes placed in the new treatment regimens for tuberculosis (TB), with the emergence of rifampicin were partially shaded by the side effects which were frequently recorded, able to impose temporary or definitive abandonment of this tuberculostatic.

## Case report

The patient to be presented illustrates relapse of TB infection, in the context of significant comorbidities: chronic renal failure, uremic stage, in chronic hemodialysis program; primary hyperuricemia with urate nephropathy; stage II arterial hypertension with very high cardiovascular risk.

In the absence of clinical, definite biological and morphological criteria, the diagnosis of drug hepatopathy relies predominantly on causal relationship between the drug administration and the occurrence of the therapeutic accident. Non-hepatitis jaundice (liver tests normal) occurred after 2 weeks of daily tuberculosis treatment, suggesting the involvement of rifampicin in the conjugation of bilirubin and conjugated bilirubin excretion in the bile. The peculiarity of the case: In the patient presented, the fundamental mechanism behind the cholestatic syndrome consisted of: compromising the active transport of bilirubin through the liver cell, without being present the reflux of bile constituents (e.g., bile acids) in the circulation.

## Conclusion

Adverse reactions to drugs due to their frequency and harmfulness have opened a new nosology chapter in modern medicine. Adverse effects of tuberculostatic drugs affect in a negative way both the management and the dynamics of TB infection.

## Consent

Written informed consent was obtained from the patient for publication of this Case report and any accompanying images. A copy of the written consent is available for review by the Editor of this journal.

